# Subclinical cardiac structural and electrical abnormalities in fibromyalgia syndrome

**DOI:** 10.3906/sag-1912-228

**Published:** 2020-06-23

**Authors:** Ekrem AKSU, Ejder BERK, Abdullah SÖKMEN, Gülizar SÖKMEN, Enes ÇELİK

**Affiliations:** 1 Department of Cardiology, Faculty of Medicine, Kahramanmaraş Sütçü İmam University, Kahramanmaraş Turkey; 2 Department of Physical Therapy and Rehabilitation, Faculty of Medicine, Kahramanmaraş Sütçü İmam University, Kahramanmaraş Turkey

**Keywords:** Arrhythmia, electromechanical delay, fragmented QRS, myocardial performance index, fibromyalgia syndrome

## Abstract

**Background/aim:**

In the literature, there is a paucity of data about the effects of fibromyalgia syndrome (FMS) on myocardial function and electrophysiological properties of atrium and ventricles. In this study, we investigated cardiac functions and noninvasive predictors of arrhythmias in patients with FMS.

**Materials and methods:**

The study included 43 female patients diagnosed with FMS and 30 age- and sex-matched healthy subjects. The presence of fragmented QRS (fQRS) morphology, P dispersion, QT dispersion, inter- and intraatrial electromechanical delay was evaluated in the groups with 12-lead ECG and standard and tissue Doppler echocardiography.

**Results:**

Among electrocardiographic parameters, P dispersion, QT dispersion, and the ratio of presence of fQRS morphology were found to be significantly higher in the study group as compared to the control group. In lateral and septal, the ratio of the early transmitral flow velocity to the early diastolic tissue velocity (E/Em) was significantly higher in the study group. Additionally, intra- and interatrial electromechanical delay was found significantly prolonged in the study group.

**Conclusion:**

FMS is found to be associated with significant cardiac electrical alterations that may indicate the increased risk of atrial and ventricular arrhythmias in this group of patients.

## 1. Introduction

Fibromyalgia syndrome (FMS) is a chronic painful disorder of musculoskeletal system characterized by fatigue, sleep disturbances, tenderness, and pain in the muscles [1]. Its prevalence has been reported as 1–10% and usually seen in middle-aged women [2]. Although many factors have been implicated in the etiopathogenesis of the disease, it has not been fully elucidated yet. Neuroendocrine abnormalities including autonomic dysfunction were thought to play an important role in the pathogenesis of the disease [3,4]. Autonomic dysfunction characterized by sympathetic hyperactivation and/or parasympathetic dysfunction in FMS patients has been shown to be related to many cardiovascular diseases including arrhythmias, and suggested to be the cause of increased cardiovascular mortality and morbidity [5–11]. 

Early detection of the risk in terms of arrhythmia and cardiovascular disease in FMS patients is important to decrease mortality and morbidity. Many electrocardiographic and echocardiographic noninvasive parameters have been studied to predict arrhythmias likely to develop in various conditions. Among them, P dispersion (Pd) and QT dispersion (QTd) obtained from surface electrocardiography (ECG) have also been investigated in FMS patients [12,13]. Another parameter, atrial electromechanical delay (EMD) that has been defined as the temporal delay between the onset of electrical activity and the realization of force in the myocardium has been widely studied to predict the risk of atrial fibrillation in various diseases [14,15]. Recently, fragmented QRS (fQRS) morphology, a new ECG parameter indicating abnormality of ventricular depolarization, has also been studied to predict atrial and/or ventricular arrhythmias in several conditions [16,17]. In the literature, there is a paucity of data about the effect of FMS on myocardial function and electrophysiological properties of atrium and ventricles. Thus, in this study, we evaluated electrical and structural cardiac changes including Pd, QTd, atrial EMD, fQRS, and myocardial performance index (MPI) by using electrocardiography and transthoracic echocardiography equipped with tissue doppler echocardiography (TDE) in patients with FMS.

## 2. Materials and methods

### 2.1. Patient selection

The study was designed as cross-sectional cohort study. The patients applying to physical therapy and rehabilitation clinic with the complaints of widespread musculoskeletal pain, fatigue, and sleep disturbances and diagnosed with FMS according to American College of Rheumatology Criteria were assessed by Fibromyalgia Impact Questionnaire (FIQ) and 43 female patients with scores of ≥50 were included in the study [18,19]. Age-matched 30 healthy women were taken as the control group. Patients with history of hypertension, coronary artery disease, heart failure, valvular heart disease, rhythm disturbances, bundle branch blocks, electrolyte disturbances, collagen tissue disease, morbid obesity, smoking, chronic alcohol use, patients using medications affecting cardiac rhythm such as antiarrhythmic, antipsychotic, and antihistaminic drugs, and patients younger than 18 years old were not included in the study. Demographic data (age, sex), body mass index (BMI, kg/m2), arterial blood pressures, and heart rates of all participants were recorded. 

### 2.2. Laboratory investigation

Venous blood samples were taken from all participants after 12 h of fasting. Complete blood count including hemoglobin level and leukocyte, neutrophil, and thrombocyte count was measured from blood samples collected into tubes with EDTA. Biochemical parameters including fasting blood glucose, aspartate aminotransferase (AST), alanine aminotransferase (ALT), total cholesterol, triglyceride, low-density lipoprotein cholesterol (LDL-C), high density lipoprotein cholesterol (HDL-C), C-reactive protein (CRP) and thyroid stimulating hormone (TSH) were measured from blood samples after centrifugation and serum separation. 

### 2.3. Electrocardiography

**Figure 1:**
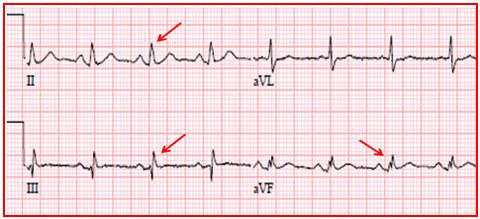
ECG sample demonstrating fragmentation of QRS in inferior derivations.

**Figure 2:**
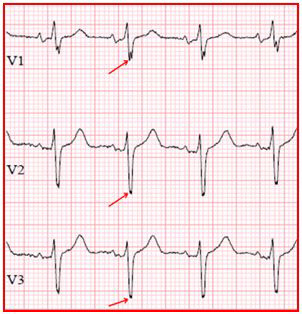
ECG sample demonstrating fragmentation of QRS in anterior derivations.

Standard 12- lead ECG samples were taken from all participants at 25 mm/s velocity on a standard ECG paper with 0.16–100 Hz filter range and 10 mm/mV voltage. Two cardiologists who were blinded to patient data measured P wave duration and QT interval from ECG samples by using a 10-fold magnification lens. P wave duration (ms) was measured from all derivations as the time between the onset and the termination point of P wave. Pd was calculated as the difference between maximum (Pmax) and minimum (Pmin) P wave durations obtained from 12 derivations. QT interval was measured from the beginning of the QRS complex to the end of the T wave. QTd was defined as the difference between the longest (QTmax) and the shortest (QTmin) QT intervals obtained from 12 derivations. fQRS morphology was defined as the presence of an additional R wave (R’), or notching in the nadir of the R wave or the S wave in the presence of normal QRS interval or the presence of > 1 R’ (fragmentation) in 2 contiguous leads corresponding to a major coronary artery territory (Figures 1 and 2) [20].

### 2.4. Transthoracic echocardiography

Echocardiographic examination was performed from left lateral decubitus position by using Vivid 7 (General Electric, Horten, Norway, 2-4 MHz phased array transducer) echocardiography machine. Echocardiographic measurements were taken together with simultaneous ECG recording by an experienced cardiologist as the average of three cardiac cycles. The measurements of left atrium (LA), aorta (Ao), left ventricular systolic (LVSD) and diastolic (LVDD) diameters, diastolic thickness of interventricular septum (IVSd) and posterior wall (PWd), left ventricular ejection fraction (LVEF), and diameter of right ventricle (RV) were obtained with M-mode echocardiography from parasternal long axis view based on the guidelines of American Society of Echocardiography. The areas of left and right (RA) atriums were obtained from apical four chamber view [21]. Mitral early diastolic (E), and late diastolic (A) velocities were measured with PW Doppler during diastole by placing sample volume to the tips of mitral leaflets, and the ratio of the early (E) to late (A) ventricular filling velocities (E/A) was calculated. 

Tissue Doppler echocardiography (TDE) was performed with transducer frequencies of 3.5–4.0 MHz using a 5-mm pulsed Doppler sample volume. Spectral Doppler signal filters were set to obtain a Nyquist limit of 15 to 20 cm/s with minimal optimal gain settings. The sweep speed was set at 50 to 100 mm/s. A single-lead ECG was recorded simultaneously during measurements. In the apical four chamber view, the sample volume was subsequently placed at the level of LV lateral mitral annulus, septal mitral annulus, and RV tricuspid annulus. The sampling window was positioned as parallel as possible to the myocardial segment of interest to obtain the optimal angle of imaging. Peak systolic (Sm), early diastolic (Em), late diastolic (Am) myocardial velocities, and isovolumic contraction time (ICT; time interval between the end of Am and the beginning of Sm), isovolumic relaxation time (IRT; time interval between the end of Sm and the beginning of Em), and ejection time (ET; time interval between the beginning and the end of Sm) were obtained from mitral and tricuspid annulus. The diastolic myocardial velocity ratio (Em/Am) for both ventricle and the ratio of the early transmitral flow velocity to the early diastolic tissue velocity (E/Em) for left ventricle were calculated. The myocardial performance index (MPI) was calculated using the formula of (ICT + IRT) / ET for both ventricles. Time intervals from the onset of P wave on the surface ECG to the beginning of the A wave (PA) representing atrial conduction were obtained from lateral mitral annulus, septal mitral annulus, and tricuspid annulus and named PA lateral, PA septum, and PA tricuspid, respectively. The difference between PA lateral and PA tricuspid was defined as interatrial EMD (PA lateral–PA tricuspid), the difference between PA lateral and PA septum was defined as intraatrial EMD (PA septum–PA tricuspid).

### 2.5. Assessment of functional status

FIQ was used to assess physical function and health status of the patients with FMS. High scores indicate severe involvement and low functionality level. Patients with scores of ≥50 points were included in the study. Points from 50 to 70 indicate moderate degree of involvement while 70 points or more indicate severe involvement in FMS patients [19]. 

### 2.6. Ethical approval

Informed consent was obtained from each patient before enrollment. The study was in compliance with the principles outlined in the Declaration of Helsinki and was approved by our institutional ethics committee.

### 2.7. Statistical analysis 

Data were analyzed with SPSS for Windows version 22 (SPSS Inc., Chicago, IL, United States). The Shapiro–Wilk test was used to test the normality of distribution for continuous variables. Continuous variables were expressed as means ± standard deviation and median (25% quartile–75% quartile). Categorical data were presented as numbers and percentages. Difference between groups was detected using chi-square test for categorical variables. Mean values of continuous variables were compared between groups using Student’s t-test or the Mann–Whitney U-test according to whether normally distributed or not. A P-value of <0.05 was considered statistically significant.

## 3. Results

The study included 2 groups: the study group composed of female patients with FMS (n = 43) and the control group included healthy women (n = 30). Demographic data of the two groups are given in Table 1. Systolic blood pressure and resting heart rate of the patients in the study group were significantly higher than those of the control group (P < 0.05). There was no significant difference between groups with regard to age, weight, height, body mass index, and diastolic blood pressure. 

**Table 1:** Demographic features of the groups.

	Study group(n=43)	Control group(n=30)	P-value
Age (year)	36.16 ± 6.52	34.83 ± 5.56	0.366
FIQ score	63.91 ± 11.16	-	
Weight (kg)	81.12 ± 9.99	78.10 ± 11.15	0.230
Height (m)	1.61 ± 0.07	1.62 ± 0.06	0.466
BMI (kg/m2)	28.87 ± 3.73	27.17 ± 3.46	0.054
SBP (mmHg)	124.65 ± 10.54	116.67 ± 9.86	0.002*
DBP (mmHg) median (Q1-Q3)	75.00 (70.00-80.00)	70.00 (65.00–80.00)	0.104†
Heart rate (beat/m)	75.74 ± 10.28	71.20 ± 8.31	0.041*†

FIQ; Fibromyalgia impact questionnaire, BMI; Body mass index, SBP; Systolic blood pressure, DBP: Diastolic blood pressure. Independent samples t-test; †

Laboratory findings of the groups are shown in Table 2. Although being within the normal range, fasting blood glucose, total cholesterol, triglyceride, LDL cholesterol, ALT, leukocyte, neutrophil, and CRP were found to be higher significantly in the study group as compared to the control group (P < 0.05). HDL-C and TSH, in spite of being within normal reference limits, were significantly lower in the study group (P < 0.05). 

**Table 2:** Laboratory findings of the groups.

	Study group(n = 43)	Control group(n = 30)	P-value
FBG (mg/dL)	92.00 ± 9.95	87.00 ± 8.75	0.030*
Total cholesterol (mg/dL)	179.77 ± 43.28	160.20 ± 32.27	0.039*
Triglyceride (mg/dL) median (Q1-Q3)	140.00 (99.00–173)	86.00 (62.25–111.00)	<0.001*†
HDL-C (mg/dL) median (Q1-Q3)	42.00 (35.00–47.00)	57.50 (46.75–65.25)	<0.001*†
LDL-C (mg/dL)	123.37 ± 27.57	99.60 ± 34.01	0.002*
AST (UI/L)	18.04 ± 4.21	17.29 ± 2.95	0.747
ALT (UI/L) median (Q1-Q3)	17.00 (14.60–19.00)	14.10 (11.85–18.08)	0.010*†
CRP (mg/L) median (Q1-Q3)	3.23 (3.02–7.07)	3.02 (3.02–3.55)	0.012*†
TSH (mIU/L) median (Q1-Q3)	1.63 (1.10–2.12)	2.35(1.57–3.49)	0.011*†
Hemoglobin (g/dL)	12.59 ± 1.29	12.75 ± 1.14	0.606
Leukocyte (/mL) median (Q1-Q3)	7.86 ± 1.98	6.68 ± 1.23	0.003*
Neutrophil (/mL)	4.44 ± 1.29	3.76 ± 0.89	0.011*
Thrombocyte (/mL)	314.738 ± 87.193	292.214 ± 52.626	0.183

FBG: Fasting blood glucose, HDL-C: High-density lipoprotein cholesterol, LDL-C: Low-density lipoprotein cholesterol , AST: Aspartate aminotransferase, ALT: Alanin aminotransferase, CRP: C-reactive protein, TSH: Thyroid-stimulating hormone.Independent samples t-test; †

Standard echocardiographic measurements of the groups are given in Table 3. IVSd, PWd, areas of LA and RA, and diameters of RA and RV were significantly higher, and LVEF, E, and E/A ratio were significantly lower in the study group as compared to the control group (P < 0.05). 

**Table 3:** Standard echocardiographic data of the groups.

	Study group(n = 43)	Control group(n = 30)	P-value
LVDD (mm)	42.72 ± 2.87	41.60 ± 2.63	0.090
LVSD (mm)	29.05 ± 2.65	28.70 ± 3.61	0.638
IVSd (mm) median (Q1–Q3)	11.00(10.00–12.00)	9.00 (8.00–10.00)	<0.001*†
PWd (mm) median (Q1–Q3)	10.00 (9.00–11.00)	8.00 (7.00–9.00)	<0.001*†
LVEF (%)	64.93 ± 2.45	67.20 ± 1.75	<0.001*
LA, diameter (mm)	29.77 ± 3.83	29.07 ± 2.75	0.393
RA, diameter (mm)	31.44 ± 3.06	29.60 ± 4.61	0.044*
RV (mm) median (Q1–Q3)	28.00(26.00–30.00)	27.00(25.00–29.00)	0.048*†
LA, area (mm2)	14.00 ± 1.21	13.32 ± 1.29	0.024*
RA, area (mm2)	12.61 ± 1.11	11.81 ± 0.96	0.002*
TAPSE (mm)	29.28 ± 2.93	29.17 ± 2.96	0.873
E (cm/s)	75.16 ± 15.21	81.13 ± 10.06	0.047*
A (cm/s) median (Q1–Q3)	67.00(57.00–79.00)	60.00 (57.25–69.25)	0.183†
E/A Ratio	1.15 ± 0.29	1.34 ± 0.24	0.005*

LVDD: Left venricular diastolic diameter, LVSD: Left ventricular systolic diameter, IVSd: Diastolic thickness of interventricular septum, PWd: Diastolic thickness of posterior wall, LVEF: Left ventricular ejection fraction, LA: Left atrium, RA: Right atrium, TAPSE: Tricuspid annular plane systolic excursion, E: Early diastolic velocity, A: Late diastolic velocity.Independent samples t-test; †Mann–Whitney U-test;

TDE measurements of the groups are shown in Table 4. Among the parameters of atrial electromechanical conduction PA lateral, PA septum, interatrial EMD, and intraatrial EMD were found to be prolonged significantly in the study group (P < 0.001, P = 0.022, P < 0.001, and P < 0.001, respectively). Additionally, lateral E/Em and septal E/Em were found to be higher, and septal Em/Am, lateral Em/Am, and tricuspid Em/Am were found to be lower in the study group as compared to the control group (P < 0.05). 

**Table 4:** Tissue Doppler echocardiographic data of the groups.

	Study group(n = 43)	Control group(n = 30)	P-value
Lateral Sm (cm/s)	9.60 ± 3.05	10.33 ± 1.97	0.254
Lateral Em (cm/s)	12.00 ± 3.38	15.63 ± 3.49	<0.001*
Lateral Am (cm/s)	10.81 ± 3.27	9.33 ± 2.60	0.043*
PA lateral (ms) median (Q1–Q3)	75.00 (72.00–83.00)	68.00 (66.25–72)	<0.001*†
Septal Sm (cm/s)	8.65 ± 2.10	9.67 ± 1.86	0.037*
Septal Em (cm/s)	10.00 ± 2.39	13.27 ± 2.68	<0.001*
Septal Am (cm/s)	9.77 ± 2.07	9.17 ± 1.64	0.189
PA septum (ms) median (Q1–Q3)	64.00 (59.00–68.00)	61.00 (56.50–62.25)	0.022*†
Tricuspid Sm (cm/s)	13.93 ± 3.54	14.20 ± 2.41	0.718
Tricuspid Em (cm/s)	14.05 ± 3.53	16.33 ± 2.95	0.005*
Tricuspid Am (cm/s)	16.81 ± 3.98	14.07 ± 4.26	0.006*
PA tricuspid (ms) median (Q1–Q3)	55.00 (50.00–61.00)	52.50 (48.75–54.75)	0.070†
Interatrial EMD (ms) median (Q1–Q3)	22.00 (19.00–25.00)	13.00 (12.00–18.00)	<0.001*†
Intraatrial EMD (ms) median (Q1–Q3)	9.00 (8.00–11.00)	6.00 (4.00–7.25)	<0.001*†
Lateral MPI	0.53 ± 0.10	0.54 ± 0.15	0.712
Septal MPI	0.55 ± 0.09	0.55 ± 0.10	0.901
RV MPI	0.59 ± 0.10	0.56 ± 0.11	0.213
LV global MPI	0.55 ± 0.07	0.54 ± 0.10	0.681
Lateral E/Em median (Q1–Q3)	6.36 (4.79–8.44)	5.00 (4.19–6.11)	0.013*†
Septal E/Em median (Q1–Q3)	7.90 (6.58–8.50)	6.18 (5.21–6.86)	0.001*†
Lateral Em/Am	1.21 ± 0.51	1.72 ± 0.63	<0.001*
Septal Em/Am	1.07 ± 0.36	1.52 ± 0.48	<0.001*
Tricuspid Em/Am	0.87 ± 0.24	1.27 ± 0.43	<0.001*

E: Early diastolic velocity of mitral inflow, Sm: Systolic velocity of myocardium, Em: Early diastolic velocity of myocardium, Am: Late diastolic velocity of myocardium, PA: Time interval from the onset of P wave to the beginning of the A wave, EMD: Electromechanical delay, interatrial EMD: PA lateral–PA tricuspid, intraatrial EMD: PA septum–PA tricuspid, RV: Right ventricular, LV: Left ventricular, MPI: Myocardial performance index.Independent samples t-test; †Mann–Whitney U-test;

Electrocardiographic measurements of the groups are given in Table 5. Heart rate calculated from ECG was higher in FMS patients (P = 0.041). Pmin and QTmin was significantly lower in the study group (P < 0.001 and P < 0.001 respectively). fQRS morphology was present in 33 (76.7%) patients of study group and in 7 (23.3%) subjects of the control group. The distribution of fragmentations on ECGs was inferior (n=20, 60.6%), anterior (n=9, 27.3%), and lateral (n=4, 12.1%) in the study group (Figures 1 and 2). On ECGs of the control group, the distribution of fQRS was inferior (n=4, 57.1%) and anterior (n=3, 42.9%). Pd, QTd, and the ratio of presence of fQRS morphology were found to be significantly higher in the study group as compared to the control group (P < 0.001, P < 0.001, and P < 0.001, respectively). 

**Table 5:** ECG measurements of the groups.

	Study group(n = 43)	Control group(n = 30)	P-value
ECG heart rate (beat/m)	75.74 ± 10.28	71.20 ± 8.31	0.041*†
Pmax (ms) median (Q1–Q3)	100.00 (90.00–100.00)	92.50 (90.00–100.00)	0.274†
Pmin (ms) median (Q1–Q3)	60.00 (50.00–70.00)	70.00 (68.75–71.25)	<0.001*†
QTmax (ms) median (Q1–Q3)	380.00 (360.00–390.00)	380.00 (367.50–390.00)	0.360†
QTmin (ms) median (Q1–Q3)	330.00 (310.00–340.00)	350.00 (340.00–360.00)	<0.001*†
Pd median (Q1–Q3)	35.00 (30.00–40.00)	22.50 (20.00–30.00)	<0.001*†
QTd median (Q1–Q3)	50.00 (40.00–60.00)	30.00 (30.00–30.00)	<0.001*†
fQRS, present n (%)	33 (76.7)	7 (23.3)	<0.001*‡

Pmax: Maximum P wave duration, Pmin: Minimum P wave duration, Pd: P dispersion, QTmax: Maximum QT interval, QTmin: Minimum QT interval, QTd: QT dispersion, fQRS: Fragmented QRS.Independent samples t-test; †Mann–Whitney U-test; ‡

## 4. Discussion

Although there are studies evaluating the effects of FMS on cardiovascular function, there is a paucity of data about the electrical alterations of conduction that may predict atrial and/or ventricular arrhythmias in FMS patients [6,9,12]. To the best of our knowledge, this is the first study evaluating fQRS morphology and inter- and intraatrial EMD in addition to ventricular functions in FMS.

Although it has not been clarified yet, central and peripheral nervous system disorders, hormonal and immunological abnormalities, genetic susceptibility, and psychiatric factors are supposed to play a role in etiology and pathogenesis of FMS [1,22]. Chronic widespread musculoskeletal pain, tender points, sleep abnormalities, and exercise intolerance depending on autonomic dysfunction constitute the clinical picture of FMS [23,24]. Numerous studies have been conducted revealing the relationship between increased cardiovascular risk and FMS. In these studies, sedentary life style due to limitation of movement, increased prevalence of metabolic syndrome, nondipper type blood pressure pattern associated with sleep disturbance, high blood pressure levels, high resting heart rate, and decreased heart rate variability are reported as the main causative factors for increased cardiovascular mortality and morbidity [3,4,9,25–28]. In our study, we investigated cardiovascular risks based on arterial blood pressure, heart rate, BMI, cholesterol levels, and CRP. Although systolic blood pressure and heart rate were within normal limits in all participants, they were significantly higher in FMS patients as compared to the control group. Furlan et al. reported that increased sympathetic and decreased parasympathetic activity caused an increase in resting blood pressure and heart rate [23]. We thought that similar results observed in our study might be clinical reflection of increased sympathetic and decreased parasympathetic activity in FMS. 

One of the reasons for increased cardiovascular risk in FMS is hypercholesterolemia. It has been shown that cholesterol level is positively correlated with the severity of the disease [29,30]. In compliance with the literature, fasting blood glucose, total cholesterol, LDL cholesterol, and triglyceride levels were significantly higher, and HDL-C cholesterol level was significantly lower in FMS patients. The mechanism underlying the increase in fasting blood glucose and cholesterol levels has not been explained clearly. Exercise intolerance and sedentary life style may have adverse effects on metabolic parameters of the patients. Additionally, insulin resistance associated with metabolic syndrome may contribute to abnormalities in these parameters. 

Standard echocardiographic measurements were found to be within normal limits in both groups. However, IVSd, PWd, diameters, and areas of left and right atrium were significantly higher, and E/A ratio was significantly lower in FMS patients. These subclinical cardiac alterations may be explained by increased cardiac afterload developing as a result of activation of renin angiotensin system triggered by the increased sympathetic activity and may indicate the tendency to develop diastolic dysfunction and other cardiovascular abnormalities. 

Previous studies revealed conflicting results about diastolic function of the left ventricle in FMS [10,31]. In our study, TDE parameters were used to evaluate diastolic function. Em/Am obtained from lateral, septal, and tricuspid annulus was significantly lower, and E/Em obtained from these three regions was significantly higher in FMS group as compared to control group. Based on our findings we cannot claim that FMS causes diastolic dysfunction, but these results may suggest that there is a tendency to develop diastolic dysfunction in FMS patients. Diverse results of previous studies may be explained by the difference in severity of the disease and methodological differences. Increased blood pressure as a result of autonomic dysfunction (increased sympathetic, decreased parasympathetic activity) may be the cause of alterations in diastolic parameters of FMS patients.

Atrial fibrillation (AF) is the most common arrhythmia encountered in clinical practice, and associated with significant mortality and morbidity due to hemodynamic impairment and thromboembolic events. Impaired atrial conduction is an important step in the pathophysiology of AF. Atrial conduction times can be evaluated with both invasive (electrophysiological study) and noninvasive (Pd on ECG and atrial EMD on echocardiography) methods [12,14,32,33]. Recently, atrial EMD has been extensively used to predict the development of atrial fibrillation. It has been shown that impaired atrial conduction is an independent and strong predictor for development and recurrence of AF, and TDE is a useful and reliable technique to evaluate atrial electromechanical properties [34,35]. Prolongation of inter- or intraatrial EMD and heterogeneous progression of sinus impulses are electrophysiological features of atrium prone to AF [32]. It has been shown that atrial EMD is prolonged in various chronic diseases, but no study has been conducted to evaluate atrial EMD in FMS [14,15]. In our study, both inter- and intraatrial EMD was found to be prolonged significantly in FMS patients. Increased sympathetic activity, and enlargement of atriums may contribute to prolongation of atrial EMD which may predict long term risk of development of atrial fibrillation and associated cardiovascular mortality and morbidity in FMS patients. 

In this study, we also evaluated Pd and QTd, cheap and easily available electrocardiographic parameters indicating heterogeneous distribution of signals through atria and ventricles, respectively. It is known that increased Pd predicts development of AF while QTd is related with ventricular arrhythmias [33,36]. Previous studies investigated Pd and QTd in FMS patients and reported no significant difference as compared to the control group. In contrast, we found that both Pd and QTd were significantly increased in FMS patients as compared to the control group. Increased Pd in addition to prolonged atrial EMD in our study supports the suggestion of increased long-term risk of AF development in FMS patients. Moreover, increased QTd in FMS patients may be related to increased risk of ventricular arrhythmias and resultant increase in cardiovascular mortality and morbidity in FMS. 

Recently, the presence of fQRS morphology in various diseases affecting cardiovascular system has been found to be related with increased mortality and morbidity [16,17]. fQRS morphology has been thought to develop as a result of heterogeneous depolarization of ventricular myocardium caused by ischemia, or fibrosis [37]. In our study, the presence of fQRS morphology was significantly more frequent in FMS patients as compared to the control group. One of the reasons for this finding may be the significant difference in systolic blood pressure of FMS patients as compared to control group. Previous studies have reported that fQRS may be a sign of chronic and continuous pressure overload, especially due to increased systolic blood pressure that leads to pathological fibrosis in the presence or even absence of left ventricular hypertrophy. Additionally, increased interventricular septal thickness and left atrial diameter in FMS patients may also be related to the presence of fQRS in accordance with previous studies [38,39]. 

There are some limitations to the present study. The first limitation is relatively small number of patients with FMS. Another significant limitation of the study is the lack of Holter ECG monitoring to document the relationship between electrical abnormalities and arrhythmias. Another limitation is that it is not known whether the patients are dipper or nondipper. Moreover, detection of myocardial fibrosis and its relation to electrical abnormalities was not shown by cardiac magnetic resonance imaging. Finally, long term follow-up of the patients could not be done to see the future arrhythmic episodes and their relationship with prolonged EMD and/or fQRS morphology since it is a cross-sectional study.

In conclusion, this study revealed subclinical cardiac electrical alterations such as increased Pd and QTd, prolonged inter- and intraatrial EMD and increased frequency of fQRS morphology which may suggest increased risk of atrial and ventricular arrhythmias in FMS patients. However, further studies with larger populations and longer follow-up periods are needed to support our findings, to detect the prognostic value of these electrical alterations, to assess their persistency, and to document their relationship with arrhythmias and long term cardiovascular complications.

## Conflict of interests

The authors report no conflicts of interests.

## Funding

The authors received no financial support for the research and/or authorship of this article.
